# Sous‐Vide Technique as an Alternative to Traditional Cooking Methods for the Technological Quality of Marinated Chicken at Industrial Scale

**DOI:** 10.1111/1750-3841.70912

**Published:** 2026-02-22

**Authors:** Karem Muraro, Rogério Luis Cansian, Geciane Toniazzo Backes, Eunice Valduga

**Affiliations:** ^1^ Department of Food Engineering URI Erechim Erechim Rio Grande do Sul Brazil

## Abstract

This study evaluated the effects of the water immersion sous‐vide technique, applied at industrial scale, on the technological and microbiological quality of marinated chicken breast fillets. Two vacuum‐packaged thermal treatments were applied: T1 (65°C for 150 min) and T2 (65°C/75°C for 200 min), along with respective non‐vacuum controls (C1 and C2). Initially, the thermal process was validated through the determination of the coldest point and the calculation of accumulated lethality (*F*_cal_), which exceeded the minimum required value (17.7 min), ensuring microbiological safety. Samples were evaluated for physicochemical composition, texture profile, and stability during 45 days of refrigerated storage (≤ 3°C). Treatment T1 demonstrated superior moisture retention (70.84%), reduced syneresis, and a softer texture (hardness: 20.76 N/cm^2^; chewiness: 11.30 N·mm), whereas T2 was characterized by distinct instrumental color parameters and a more homogeneous texture profile. All treatments exhibited low water activity (aw; 0.9825–0.9855), stable pH, and peroxide index below 0.5 mEq O_2_/kg. Microbiological analyses confirmed the absence of *Salmonella* spp. and undetectable levels of *Escherichia coli*, *Clostridium perfringens*, *Staphylococcus aureus*, and *Listeria monocytogenes* throughout 45 days of storage, in accordance with legal limits. Multivariate analysis revealed significant correlations between texture and composition, highlighting the positive influence of vacuum packaging and thermal regime on final product quality. It is concluded that water immersion sous‐vide is an effective technique for producing marinated chicken meat with enhanced technological quality, extended stability, and microbiological safety, representing a promising alternative for the ready‐to‐eat food industry.

**Practical Applications:**

The industrial‐scale sous‐vide cooking enhances the technological quality of marinated chicken, resulting in a uniform texture and extended shelf life while ensuring microbiological safety. Compared to traditional cooking methods, this approach minimizes moisture loss and color deterioration, increases product consistency, and reduces the need for additives, resulting in cleaner labels.

In addition, sous‐vide offers relevant economic and energetic advantages for industry: precise temperature control can lower energy consumption, higher moisture retention improves yield, and extended stability reduces product waste and enhances distribution efficiency. These factors make sous‐vide a competitive and cost‐effective alternative for ready‐to‐eat poultry products.

## 1 Introduction

The growing demand for healthier, more sustainable, and convenient foods, with extended shelf life and preservation of nutritional and sensory attributes, has driven the food industry to adopt technological innovations that meet these requirements, reduce waste, and align with contemporary consumption trends (Guiné et al. 2020). In this context, techniques that optimize food preparation and presentation have gained prominence, especially in meat‐based products widely used in ready‐to‐eat meals, convenience foods, and delicatessen items (Wereńska 2024).

Innovation in meat processing plays a key role in diversifying the food supply, particularly within the growing ready‐to‐eat segment. Among the emerging alternatives, the sous‐vide cooking method has become a promising approach for the food industry (Kathuria et al. 2022). The term “sous‐vide,” derived from French meaning “under vacuum,” refers to the cooking of vacuum‐sealed foods in heat‐stable plastic bags, subjected to precisely controlled temperatures, typically in a water bath. The process is characterized by slow cooking at relatively low temperatures, followed by rapid cooling (Fellows 2019; Teeratantikanont 2013; Baldwin 2012). The temperatures generally range from 70°C to 95°C, significantly lower than those used in conventional sterilization methods such as canning (130°C–150°C for a few seconds) (Damodaran and Parkin 2019; Sebastiá et al. 2010; Stringer and Metris, 2018).

In Brazil, although there is no specific regulation for sous‐vide cooking, ready‐to‐eat foods must undergo thermal treatments that ensure food safety, such as reaching 75°C at the coldest point or applying equivalent time–temperature combinations effective in reducing pathogenic microorganisms to safe levels (Brazil 2022a). These conditions can be precisely controlled using water bath systems or industrial‐scale equipment such as steam, convection, or combi ovens (Roldán et al. 2015; Baldwin 2012).

Among the main benefits of the sous‐vide technique are the gradual denaturation of proteins, allowing precise control of texture, and vacuum packaging, which minimizes moisture loss through osmosis. In addition, the method helps preserve water‐soluble vitamins sensitive to oxidation, increases mineral bioaccessibility, reduces the formation of free radicals, and inhibits oxidative reactions. These factors contribute not only to maintaining sensory and nutritional quality but also to microbiological safety by limiting the growth of aerobic bacteria and preventing recontamination, resulting in extended shelf life and reduced need for preservatives, as well as potential savings in materials, labor, and storage (Thathsarani et al. 2022; Ayub and Ahmad 2019; Kathuria et al. 2022).

In recent years, several studies have examined the effects of sous‐vide processing on the physical, biochemical, and microbiological properties of meats. Evidence indicates improvements in texture, nutritional value, and digestibility in beef (S. Sun et al. 2019; Uttaro et al. 2019), pork (Hwang et al. 2019; Joung et al. 2018), lamb (Ortuño et al. 2021; Ruiz‐Carrascal et al. 2019), chicken (Chian et al. 2021; Haghighi et al. 2021; Park et al. 2020; Zhu et al. 2018), and turkey (Bıyıklı et al. 2020).

Despite the increasing application of sous‐vide in food services and scientific research, studies evaluating its implementation at industrial scale remain scarce, especially for marinated meat products of greater thickness, such as chicken breast fillets. Most existing research is limited to small cuts under laboratory conditions and does not address critical variables in large‐scale processing, such as cold‐spot determination, optimal time–temperature parameters, and validation of thermal lethality under real production conditions.

In this context, the present study investigates, for the first time, the application of industrial‐scale water immersion sous‐vide cooking in marinated chicken breast fillets, with a focus on microbiological safety, technological quality, and product stability during refrigerated storage. In addition, the study includes physicochemical and textural analyses, providing relevant data to support the validation and potential adoption of this technique by the poultry industry as an alternative to conventional cooking methods.

Therefore, the aim of this study was to validate the water immersion sous‐vide cooking process for marinated chicken breast fillets at industrial scale, assessing the effects of the thermal treatment on technological quality and microbiological stability of the product stored under refrigeration (≤ 3°C).

## 2 Materials and Methods

The chicken breast fillets used in this study were obtained from a federally inspected poultry processing plant in southern Brazil. All experimental procedures were developed and performed at this processing plant under routine industrial conditions and following the plant's standard operating procedures.

Initially, fillet thickness (measured at the thickest point) was recorded prior to tumbling in order to define appropriate time and temperature parameters for the thermal treatment. Samples with thickness ranging from 40 to 50 mm and weighing 280 to 320 g were selected, totaling 80 kg per treatment.

The base marination formulation consisted of 88.5% chicken breast, 10% water, 1% sodium chloride (NaCl), and 0.5% modified corn starch, based on the fillet weight. The tumbling process was performed under vacuum using a Tumbler Maxmac (model Tumbles, 70 L capacity) at 15 rpm for 30 min to promote massage and uniform brine incorporation (Delles and Xiong 2014).

**FIGURE 1:**
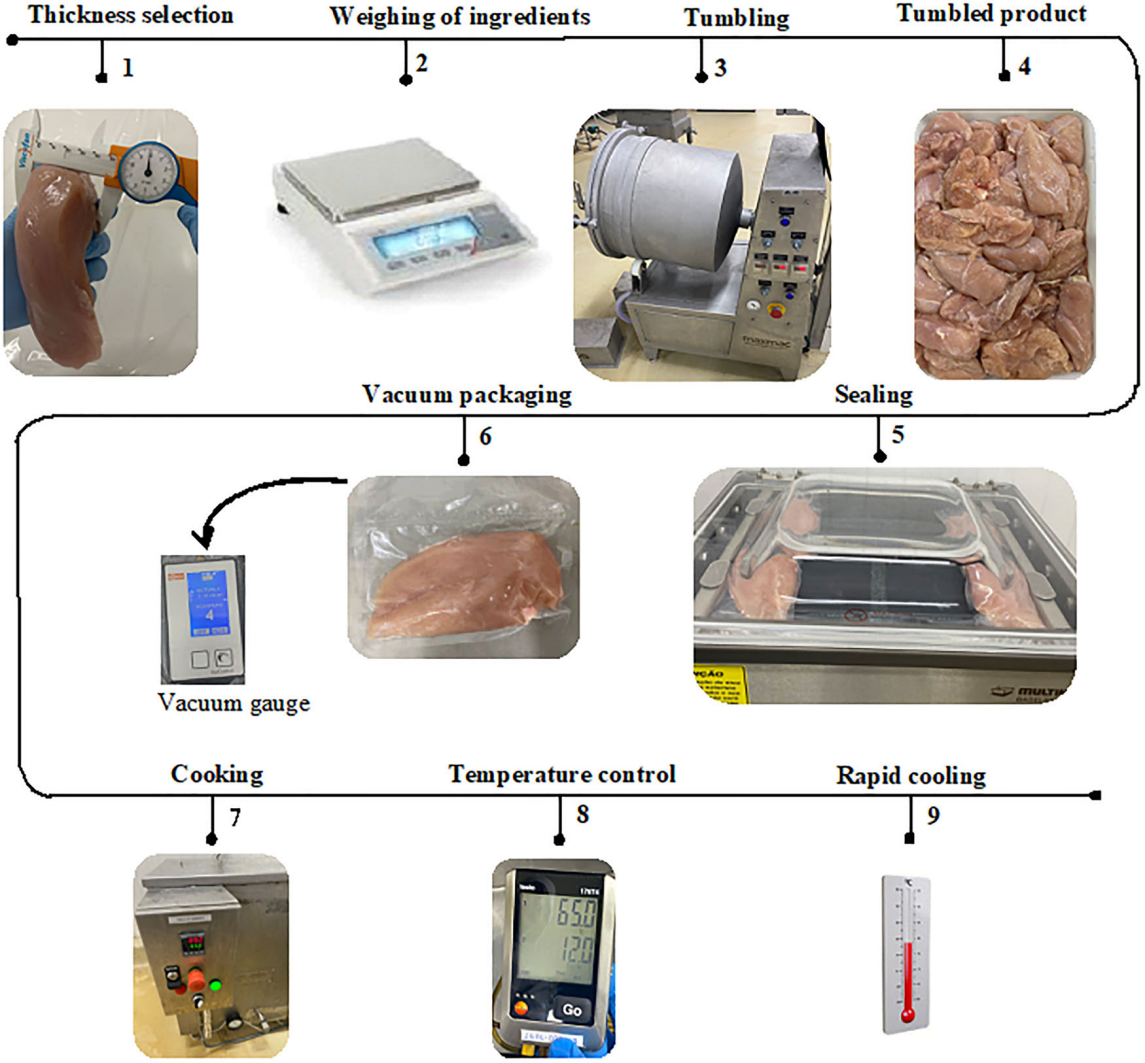
Steps of the process for preparing marinated chicken breast fillet subjected to heat treatment using the sous‐vide technique.

Following tumbling, each fillet was individually vacuum‐sealed using a Multivac Baseline P 360 sealer. Packaging material was a multilayer film composed of high‐density polyethylene terephthalate (PET), low‐density polyethylene (LDPE), linear low‐density polyethylene (LLDPE), and polyamide (PA), with dimensions of 220 mm × 200 mm and a thickness of 180 µm. The oxygen transmission rate (OTR) of the packaging was 56.496 cm^3^/m^2^/day at 23°C and 0% relative humidity, according to ASTM D 3985‐10 (ASTM‐INTERNATIONAL 2010). For the vacuum treatments (T1 and T2), vacuum was applied to a pressure of 45 mbar, as verified with a digital vacuum gauge (VacControl, Busch). Control samples were packed using the same material, but without vacuum. The processing steps from selection to packaging are summarized in Figure 1, following the criteria established by Brazilian Normative Instruction No. 17/2018 (Brazil 2018).

### 2.1 Cooking Process

#### 2.1.1 Thermal Process Validation

**FIGURE 2:**
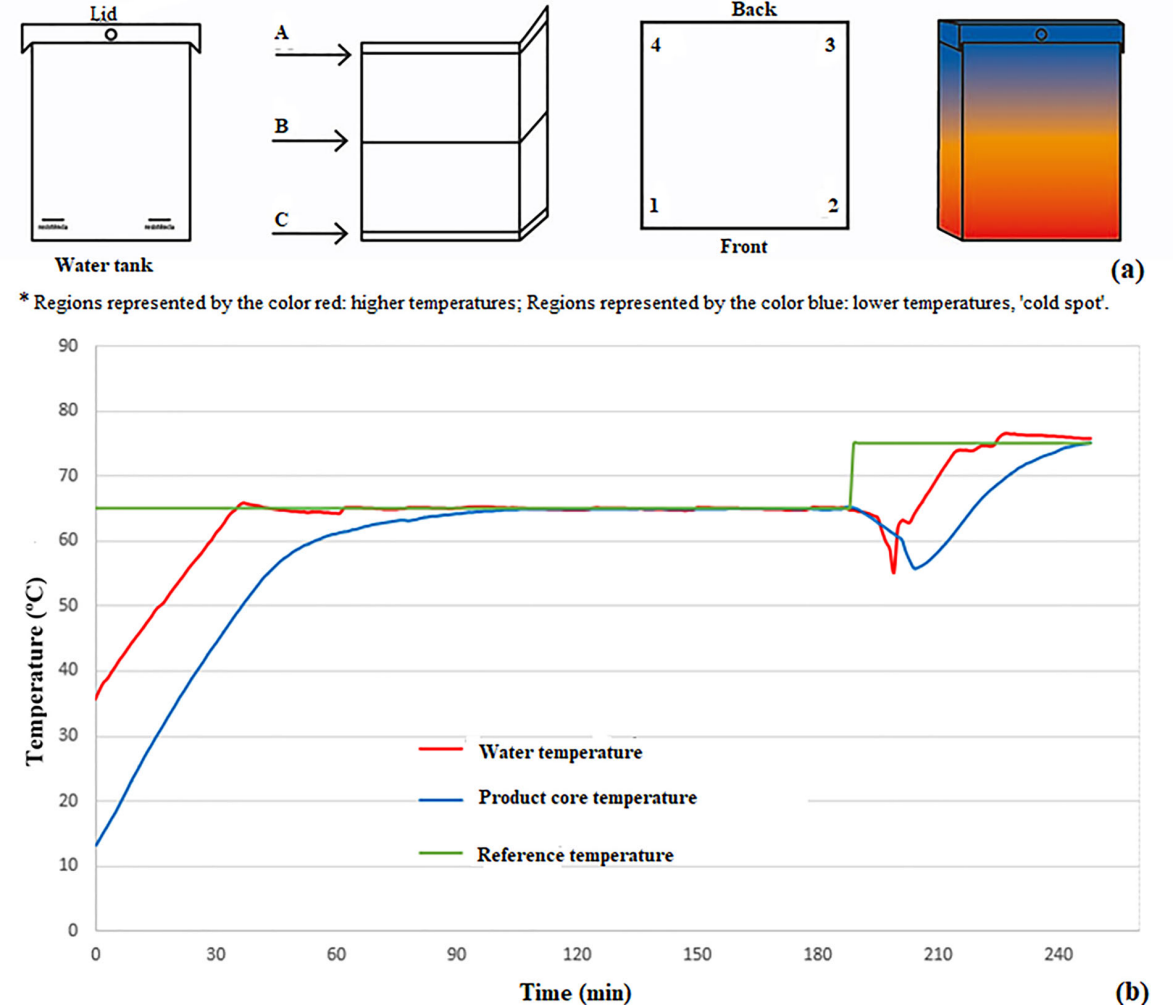
Schematic of sensor placement and heat distribution in the water immersion cooking tank (a) and time–temperature profile of the cooking cycles for marinated chicken breast fillets in a cooking tank treated with water immersion (b).

Prior to thermal treatment, heat distribution within the water immersion tank was evaluated to identify the coldest point in the system. This was monitored using a data logger (model T176T4, Testo, Campinas, Brazil; operating range: −100°C to 1000°C), following the method described by Santos Filho and Penna (2003), adapted for 1‐min intervals. The device was equipped with four probes positioned at various heights (A, B, C) and locations (1, 2, 3, 4) within the tank (Figure 2a).

Validation of the thermal process, essential for ensuring microbiological safety and shelf life, was based on the calculation of thermal lethality, expressed as the *F* value (or *F*_0_), which represents microbial log reduction during heating (Berteli et al. 2013). For ready‐to‐eat refrigerated products, a 6‐log reduction is typically required. The *F* value was calculated based on the thermal resistance of the target microorganism, which varies according to the type of food (Stumbo 1965).

The thermal lethality calculation requires the *D* value (time needed for a 1‐log microbial reduction at a reference temperature) and the *z* value (temperature increase required to reduce the *D* value by one log). These parameters were obtained from literature data specific to each microorganism (Aryani et al. 2015). In this study, *Streptococcus* D (*Enterococcus* family) was selected as the reference organism, commonly used for validating thermal processing of ready‐to‐eat foods (Feiner 2006). The reference values used were: *T*_ref_ = 70°C, *D*_ref_ = 2.95 min, and *z* = 10°C.

Lethality rate (*L*) was calculated using Equation 1, based on the difference between the reference temperature (*T*_ref_) and the actual process temperature (T1):

(1) $$D_{T2}/D_{T1}=10^{\left(T1-T2\right)/Z}$$

where *D*_T2_/*D*_T1_ = *L*.

The reference lethality value (*F*_ref_) was calculated from the initial (*N*_0_) and final (*N*) microbial loads expected in the product, as shown in Equation 2:

(2) $$F_{\mathrm{ref}}=D_{\mathrm{ref}}\left(\log N_{0}-\log N\right)$$

where *D*_ref_ is the decimal reduction time, *N*_0_ is the initial pathogen load per gram of raw product, and *N* is the maximum number of surviving bacteria after processing.

To verify process efficacy, the accumulated lethality (*F*_cal_) was calculated as the sum of lethal contributions throughout the cooking period, using Equation 3:

(3) $$F_{\mathrm{cal}}={\left(L_{1}+L_{2}=L_{3}\text{…}\text{…}..L_{n-1}\right)}^{*}\Delta t$$

where *F*_cal_ is the cumulative lethal effect over each time interval Δ*t*.

The process was considered validated when *F*_cal_ ≥ *F*_ref_, confirming that the microbial load was reduced to safe levels, ensuring the microbiological stability of the refrigerated product.

#### 2.1.2 Thermal Process Validation

Thermal treatments were carried out via water immersion (water bath), as illustrated in Steps 7, 8, and 9 of Figure 1. Sous‐vide samples T1 and control C1 (non‐vacuum‐packed) were cooked in a thermostatically controlled tank set to 65°C and monitored until the geometric center (coldest point) reached 65°C. Once achieved, the samples remained in the bath for an additional 150 min, maintaining core temperature at 65°C. The total time with the core temperature between 55°C and 65°C was approximately 165 min. After cooking, the products were immediately transferred to a freezing chamber at −18°C until the internal temperature reached 3°C, which occurred within 90 min.

Treatment T2 and its control C2 were initially subjected to the same conditions as T1 (65°C for 150 min). Subsequently, the water bath temperature was increased to 75°C and maintained until the core temperature of the fillets reached 75°C, which took approximately 50 min. The total duration with internal temperature between 55°C and 75°C was approximately 200 min. After cooking, samples were also cooled to 3°C at the core in a −18°C chamber, within 90 min.

After sous‐vide processing, samples were evaluated for physicochemical parameters (moisture, protein, lipid content, and cooking loss) and texture profile (shear force, hardness, adhesiveness, cohesiveness, and chewiness).

#### 2.1.3 Storage Stability

The stability of the thermally processed samples was evaluated over a 45‐day refrigerated storage period (≤ 3°C). The following parameters were monitored: titratable acidity, pH, water activity (aw), syneresis, peroxide index, and color parameters.

Microbiological assessment included total aerobic mesophilic counts in raw material, post‐cooking samples, and throughout storage. Specific analyses were performed for *Staphylococcus aureus*, *Escherichia coli*, *Salmonella* spp., *Clostridium perfringens*, and *Listeria monocytogenes*, both immediately after cooking and at storage intervals. All microbiological tests followed Brazilian regulatory standards for ready‐to‐eat products (Brazil 2022a), aiming to ensure food safety throughout the intended shelf life.

### 2.2 Analytical Determinations

#### 2.2.1 Physicochemical Analyses

Moisture was determined by oven drying at 105°C following ISO 1442:1997 and the Brazilian Ministry of Agriculture Standard 1.29 (Brazil 2022b).

Protein content was determined via total nitrogen analysis using the Kjeldahl method, according to ISO 1871:2009 and Brazilian Ministry of Agriculture Standard method 1.24 (Brazil 2022b). The result was converted to protein by multiplying the nitrogen content by 6.25.

Lipid content was determined according to ISO 1443:1973 and Brazilian Ministry of Agriculture Standard method 1.19 (Brazil 2022b).

Cooking loss was assessed by weighing the cooked chicken breast samples after cooling (3 ± 2°C), blotting with paper towels to remove surface moisture, and calculating the percentage difference between initial and final mass (Honikel 1998). Peroxide value was determined using ISO 3960:2017 (International Organization of Standardization 2017).

Acidity was determined according to the official method of the Adolfo Lutz Institute (IAL 2008). aw was measured using an Aqualab Series 4TE (Meter Food), calibrated with 6.0 M NaCl solution (aw = 0.76) and 8.57 M LiCl solution (aw = 0.50). Samples were previously equilibrated at 25°C.

pH was measured with a digital pH meter (DIGIMED, model pH 300 M), calibrated with pH 3.0 and 7.0 buffer solutions Brazil (Brazil 2022b). The electrode was inserted directly into the marinated, cooked chicken samples.

Color parameters were measured using a portable colorimeter (Minolta CR‐400, Konica Minolta Sensing Americas Inc., Ramsey, NJ) connected to SpectraMagic NX software. The instrument was calibrated with white and black reference standards prior to analysis. Measurements were obtained in the CIEL^*^ *a* ^*^ *b* ^*^ color space, where *L* ^*^ represents lightness, *a* ^*^ the red–green axis, and *b* ^*^ the yellow–blue axis. Chroma (*C* ^*^), or saturation, was calculated according to standard procedures (Stewart et al. 1965). Total color difference (Δ*E*) between sous‐vide and control treatments was calculated using Equation 4 (Djekic et al. 2021; Tomasevic et al. 2019):

(4) $$\Delta E=\left[{\left(\Delta L^{*}\right)}^{2}+{\left(\Delta a^{*}\right)}^{2}+{\left(\Delta b^{*}\right)}^{2}\right]1/2$$

Readings were taken at three distinct points on the surface of each marinated and cooked fillet, with superficial moisture removed using paper towels. Mean values (*n*  =  5) were used for statistical analysis (International Organization of Standardization 2024).

Syneresis was determined based on the method of Khouryieh et al. (2005), with modifications. Samples were removed from the package, and the exuded liquid was collected in a beaker. Excess surface liquid was absorbed using pre‐weighed paper towels. Syneresis was expressed as the ratio of released liquid mass to the total sample mass.

Texture profile analysis (TPA) was conducted following López‐Pedrouso et al. (2018). Chicken breast fillets were cubed into 2.0 cm cubes perpendicular to muscle fibers using a ruler for precision. A calibrated texture analyzer (TA.XT Plus, Stable Micro Systems, Surrey, UK) was used. Samples were positioned with fibers perpendicular to the probe and compressed in two cycles at 40% deformation, with a test speed of 0.50 mm/s and a 1‐s interval between compressions. Parameters assessed included hardness, adhesiveness, cohesiveness, and chewiness.

Shear force was measured on whole cooked fillets using the same texture analyzer equipped with a Meullenet‐Owens Razor Shear Blade (MORS), operating at a speed of 10 mm/s, 20 mm penetration depth, and 5 g trigger force at 22 ± 2°C (X. Sun et al. 2021; Lee et al. 2008). MORS measurements were taken at four different points in the cranial region of each fillet.

#### 2.2.2 Microbiological Analyses

*C. perfringens* (sulfite‐reducing): quantified by the pour plate method (International Organization of Standardization 2023). Coagulase‐positive *Staphylococcus*: enumerated using surface plating (AFNOR 2003). *Salmonella* spp.: presumptive detection via polymerase chain reaction (PCR) (AFNOR 2016). *L. monocytogenes*: presumptive detection via PCR (AOAC‐2004.02 2004). Total mesophilic aerobic bacteria: determined according to ISO 4833‐1:2013/Amd1 2022 (International Organization of Standardization 2013).

### 2.3 Statistical Analysis

All experiments were performed with 30 replicates per treatment. Data were analyzed by analysis of variance (ANOVA) followed by Tukey's test to compare means, using Statistica version 5.0 (Statsoft Inc., USA) at a 95% confidence level. Pearson's correlation and principal component analysis (PCA) were conducted using XLSTAT 2020 Free version.

## 3 Results and Discussion

### 3.1 Monitoring of Thermal Treatment

Figure 2b shows the temperature profile over time during the cooking process, demonstrating the system's ability to maintain stable temperatures, even at the coldest point within the water immersion tank. A brief temperature drop was observed when the tank was opened for sample collection, reducing the water temperature to approximately 55°C, while the core temperature of the product decreased less markedly. After the tank was closed, the system gradually recovered, returning to the 65°C set point in about 30 min. The temperature curves recorded both at the center of the chicken fillets and in the tank water remained close to the programmed value, confirming the high precision of the thermal controllers.

In immersion‐based cooking systems, heat transfer occurs predominantly by conduction. This process is enhanced by the high surface moisture of the food (close to 100%), which increases thermal efficiency. As reported by Baldwin (2012), such systems allow uniform heat transfer, with temperature variations typically below 0.2°C, consistent with the observations in this study.

Thermal process validation was based on the calculation of accumulated lethality (*F*_cal_), using the thermal resistance parameters of the reference microorganism *Streptococcus* D. The reference values used were: *T*_ref_  =  70°C, *D*_ref_  =  2.95 min, and *z*  =  10°C. The minimum lethality value (*F*_ref_) required to ensure a 6‐log reduction (6D) was calculated as 17.7 min.

The *F*_cal_ value represents the sum of all lethal contributions (*L*) over time intervals (∆*t*) during cooking, and is influenced by both the temperature reached and the duration of exposure. The highest *F*_cal_ values were observed for T2 and C2 treatments (Table 1), likely due to more efficient heat transfer via water immersion, particularly in vacuum‐packaged samples, which favor rapid thermal conduction toward the product's interior.

**TABLE 1:** Data on the accumulated lethality rate (*F*_cal_) and the estimated number of logarithmic reductions for chicken breast treatments using water immersion and steam oven at different temperatures.

Treatment^*^	Accumulated lethality rate *F*_cal_ (min)	Estimated log reduction
**C1**	37.44	12.69
**T1**	37.44	12.69
**C2**	93.47	31.68
**T2**	94.07	31.89

* Cooking until internal temperatures of 65°C and 75°C were reached in a water immersion tank for treatments T1 and T2, respectively. Treatments C1 and C2 were packaged without vacuum.

It is important to note that the initial heating phase contributes little to total lethality, as significant bactericidal effects only begin when the product's core temperature exceeds 55°C (Feiner 2006). From this threshold, thermal lethality begins to accumulate continuously until the cooling phase. Therefore, the effectiveness of the process is not determined solely by achieving a target temperature, but also by the time the product remains within the lethal temperature range.

Although all treatments achieved a log reduction greater than 6, this parameter alone does not fully define the overall effectiveness of the thermal process. The objective includes not only microbiological safety, but also the achievement of desirable sensory characteristics, such as juiciness and tenderness. Because thermal processes are dynamic, with temperature varying over time, lethality calculations must consider the entire thermal history (cooking curve) recorded during processing. Thus, the longer the product core remains above 55°C, the greater the accumulated lethality and, consequently, the higher the effectiveness of the treatment from a microbiological standpoint.

### 3.2 Technological Characteristics of the Product

Table 2 presents the physicochemical analysis results for marinated chicken breast fillets cooked using the water immersion sous‐vide technique. The parameters evaluated included cooking loss, moisture, protein, and lipid content. A significant difference (*p* < 0.05) was observed among treatments, with T1 showing the highest moisture retention. This result is consistent with the findings of Bıyıklı et al. (2020), who reported lower water loss (7.36%) in turkey meat processed sous‐vide at 60°C for 90 min.

**TABLE 2:** Moisture, protein, fat content, and cooking loss (CL) of marinated chicken breast fillet samples subjected to heat treatment using the sous‐vide technique and their respective controls (C).

Treatment^*^	Moisture (g/100 g)	Protein (g/100 g)	Fat (g/100 g)	CL (g/100 g)
C1	78.27^b^ ± 0.09	18.05^d^ ± 0.01	2.07^ab^ ± 0.04	1.99^ab^ ± 0.14
T1	79.50^a^ ± 0.08	20.13^b^ ± 0.01	1.83^b^ ± 0.09	1.75^b^ ± 0.05
C2	76.60^d^ ± 0.14	18.18^c^ ± 0.01	1.53^c^ ± 0.05	2.27^a^ ± 0.03
T2	77.23^c^ ± 0.09	20.27^a^ ± 0.02	1.50^c^ ± 0.08	2.05^ab^ ± 0.12

*Note*: Means ± standard deviation, followed by identical lowercase letters within a column, indicate no significant difference (*p* > 0.05, Tukey's test).

* Cooking until internal temperatures of 65°C and 75°C were reached in a water immersion tank for treatments T1 and T2, respectively. Treatments C1 and C2 were packaged without vacuum.

Treatment T2 showed greater moisture loss and liquid exudation, likely due to thermal denaturation of myofibrillar proteins, which leads to muscle fiber shrinkage (Tornberg 2005), exacerbated by the extended exposure at 75°C. During sous‐vide cooking, some sarcoplasmic proteins are also solubilized into the water (Rasinska et al. 2019), contributing to exudate formation. On the other hand, several studies have shown that sous‐vide can preserve moisture and reduce cooking loss, particularly in white meats (Haghighi et al. 2021; Park et al. 2020). Hasani et al. (2022), reported moisture values of 71.21% and 68.90% in chicken breast fillets cooked at 60°C for 120 and 180 min, respectively. Similarly, Nyam et al. (2023) observed a slight decrease in moisture content with increasing time and temperature, reporting values of 70.40% and 68.74% at 60°C for 45 and 135 min, and 69.10% and 66.85% at 70°C for the same durations.

Table 3 shows the texture profile results for fillets subjected to sous‐vide treatments and their respective non‐vacuum controls. Treatment T2 presented higher hardness and lower shear force values, suggesting an effect of the extended time/temperature combination on protein structure. Prolonged thermal exposure promotes collagen dissolution and breakdown of myofibrillar fibers, which alters meat tenderness (Roldán et al. 2015). Tenderization may also result from solubilization of connective tissue, facilitated by the moist environment of vacuum cooking (Laakkonen et al. 1970).

**TABLE 3:** Texture profile of marinated chicken breast fillet samples subjected to heat treatment using the sous‐vide technique (T) and their respective controls (C).

Treatment^*^	Shear force (N)	Hardness (N/cm^2^)	Adhesiveness (N . s)	Cohesiveness	Chewiness (N mm)
C1	6.19^ab^ ± 0.06	19.52^b^ ± 1.20	−0.1213^a^ ± 0.022	0.69^a^ ± 0.03	11.17^b^ ± 0.69
T1	6.42^a^ ± 0.12	18.50^b^ ± 0.24	−0.0957^a^ ± 0.008	0.70^a^ ± 0.01	11.30^b^ ± 1.01
C2	5.83^bc^ ± 0.05	18.94^b^ ± 0.55	−0.0944^a^ ± 0.016	0.66^b^ ± 0.02	9.87^c^ ± 0.76
T2	5.39^c^ ± 0.03	24.89^a^ ± 0.27	−0.1026^a^ ± 0.010	0.66^b^ ± 0.03	13.22^a^ ± 0.87

*Note*: Means ± standard deviation, followed by identical lowercase letters within a column, indicate no significant difference (*p* > 0.05, Tukey's test).

* Cooking until internal temperatures of 65°C and 75°C were reached in a water immersion tank for treatments T1 and T2, respectively. Treatments C1 and C2 were packaged without vacuum.

In the case of T1 and its control, the results indicated greater tenderness, likely due to the milder thermal treatment (65°C for 150 min), which weakens connective tissue and partially aggregates sarcoplasmic proteins, contributing to a softer texture (Tornberg 2005; Baldwin 2012). Nyhan et al. (2018) also observed reduced hardness in sous‐vide cooked chicken breast, especially at 60°C and 70°C for 135 min, with values around 34.63 N. Chang et al. (2023), using sous‐vide on duck breast for 90 min, reported hardness values of 34.2 N at 65°C and 34.05 N at 70°C, which are comparable to those found in this study.

For chewiness, the present study reported a value of 11.30 N·mm for treatment T1, which is similar to the value of 11.24 N·mm reported by (Kerdpiboon et al. 2019), for sous‐vide chicken breast cooked at 60°C for 5 h.

Regarding cohesiveness, a tendency for higher values was observed in treatments carried out at lower temperatures (65°C—T1). Similar results were reported by Chang et al. (2023), for duck breast (0.66) and by Hasani et al. (2022) and Park et al. (2020), who also found no significant changes in cohesiveness in chicken breast cooked sous‐vide under different time–temperature combinations.

According to Nyhan et al. (2018), texture variation in brined meats cooked sous‐vide depends on time and temperature conditions, influencing hardness, cohesion, and elasticity, but not adhesiveness. In this study, treatment T2 showed higher hardness and chewiness, whereas T1 resulted in a more tender and cohesive texture, reinforcing the importance of thermal control in determining final product quality.

All treatment samples (C1, T1, C2, and T2) tested negative for *C. perfringens*, *S. aureus*, *E. coli*, and *Salmonella* spp., meeting the legal limits for ready‐to‐eat products Brazil (Brazil 2022a).

**FIGURE 3:**
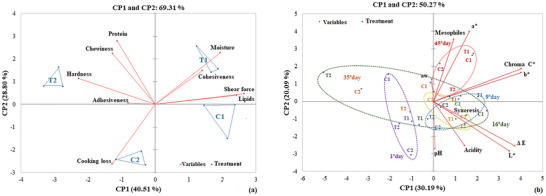
Principal component analysis (PCA) for marinated chicken breast fillet samples subjected to different heat treatments using the sous‐vide technique and their respective controls (a) and during storage at < 3°C (b), respectively.

Figure 3a shows the PCA performed using physicochemical variables and instrumental texture of marinated chicken breast fillets subjected to different sous‐vide thermal treatments. In the biplot, variables are represented by vectors; longer vectors indicate greater contribution to variance between treatments. Samples are represented by triangles, with each vertex corresponding to the mean of 10 experimental replicates.

The first principal component (PC1) explained 40.51% of the variance, and the second (PC2) explained 28.80%, totaling 69.31% of the total variability in the two‐dimensional plane. This distribution demonstrates good discriminatory capacity of the PCA between treatments.

Pearson's correlations confirmed the relationships observed in the PCA biplot. Moisture content was strongly and negatively correlated with cooking loss (*r*  =  −0.815) and positively correlated with shear force (*r*  =  0.786), and to a lesser extent, with cohesiveness (*r*  =  0.530). This suggests that higher moisture levels reduce cooking loss and enhance the structural integrity of the product.

Hardness was negatively correlated with chewiness (*r*  =  −0.763; *p* < 0.05), suggesting that harder products required less chewing work. Shear force was strongly negatively correlated with hardness (*r*  =  −0.799), reinforcing the notion that a reduction in the force needed to cut the sample is associated with greater perceived structural firmness. Shear force also correlated positively with lipid content (*r*  =  0.759), weakly with cohesiveness (*r * =  0.529), indicating that tougher samples were less appreciated by consumers.

Protein content showed a positive correlation with chewiness (*r*  =  0.601). Overall, the results indicate that texture was the most affected parameter by thermal processing conditions, particularly in T2 samples, which reached a core temperature of 75°C under vacuum. This condition led to structural changes in myofibrillar and sarcoplasmic proteins, enhancing the balance between tenderness and firmness.

### 3.3 Storage Stability

During refrigerated storage (≤ 3°C for 45 days), peroxide index values remained below 0.5 mEq O_2_/kg of fat on Days 1, 9, 16, 25, 35, and 45, indicating low levels of lipid oxidation in all products analyzed. By comparison, Bıyıklı et al. (2020) reported higher values in sous‐vide duck ribs, ranging from 1.24 to 1.56 mEq O_2_/kg depending on cooking time and temperature (60°C–75°C).

**FIGURE 4:**
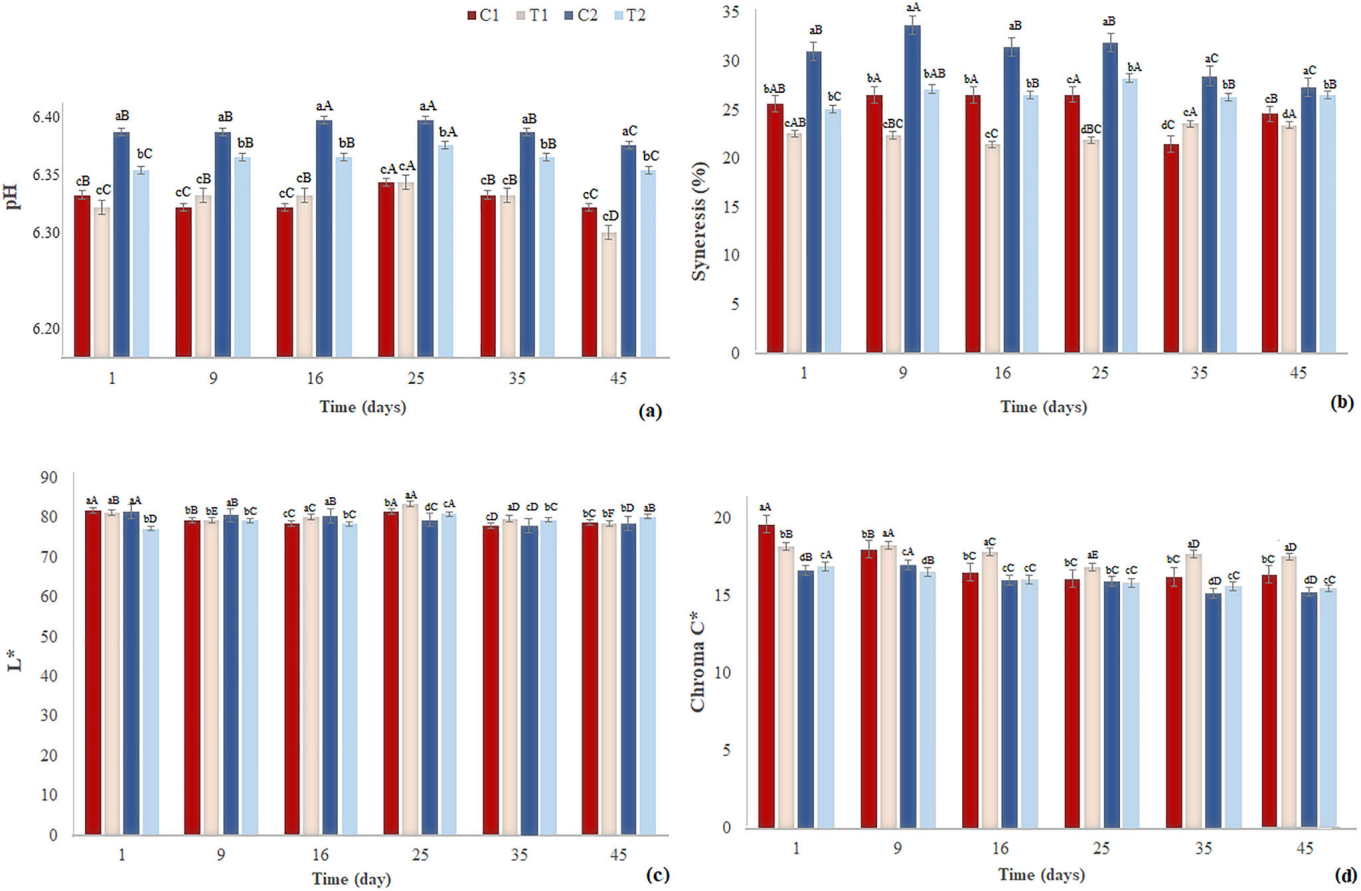
pH (a), syneresis (b), and color parameters (*L* ^*^(c) and chroma C (d)) of marinated chicken breast fillet samples subjected to sous‐vide treatment and their respective controls, during storage at ≤ 3°C.

The pH of sous‐vide chicken breast fillets was significantly affected (*p* < 0.05) by both cooking temperature and the time–temperature interaction (Figure 4a). A progressive pH increase was observed in samples subjected to higher cooking temperatures and longer times, particularly in T2 and C2 (75°C/180 min). These results are in line with Bıyıklı et al. (2020), who attributed pH elevation to protein denaturation and changes in protein surface charges.

Regarding syneresis (Figure 4b), vacuum‐packaged treatments (T1 and T2) exhibited lower fluid loss during storage compared to control groups (C1 and C2), with T1 showing the lowest values (*p* < 0.05). The higher exudate levels in non‐vacuum samples may be attributed to greater degradation of the myofibrillar structure, exacerbated by the absence of vacuum pressure and elevated temperatures. Vacuum packaging helps preserve moisture and structural integrity, minimizing water loss during storage (Rao et al. 2022; Baldwin 2012).

Water‐holding capacity (WHC) in sous‐vide‐treated meats is closely linked to pH and cooking temperature. Near the isoelectric point (pH ≈ 5.5), proteins lose their ability to retain water, increasing syneresis. Higher pH values, as observed in T1, favor WHC by limiting protein denaturation and preserving muscle structure, thereby reducing exudation during storage. aw ranged between 0.9825 and 0.9855, with no significant differences (*p* > 0.05) among treatments throughout the storage period. These findings are consistent with reports by Bıyıklı et al. (2020) and Nyam et al. (2023), who also observed aw stability in sous‐vide processed meats.

**FIGURE 5:**
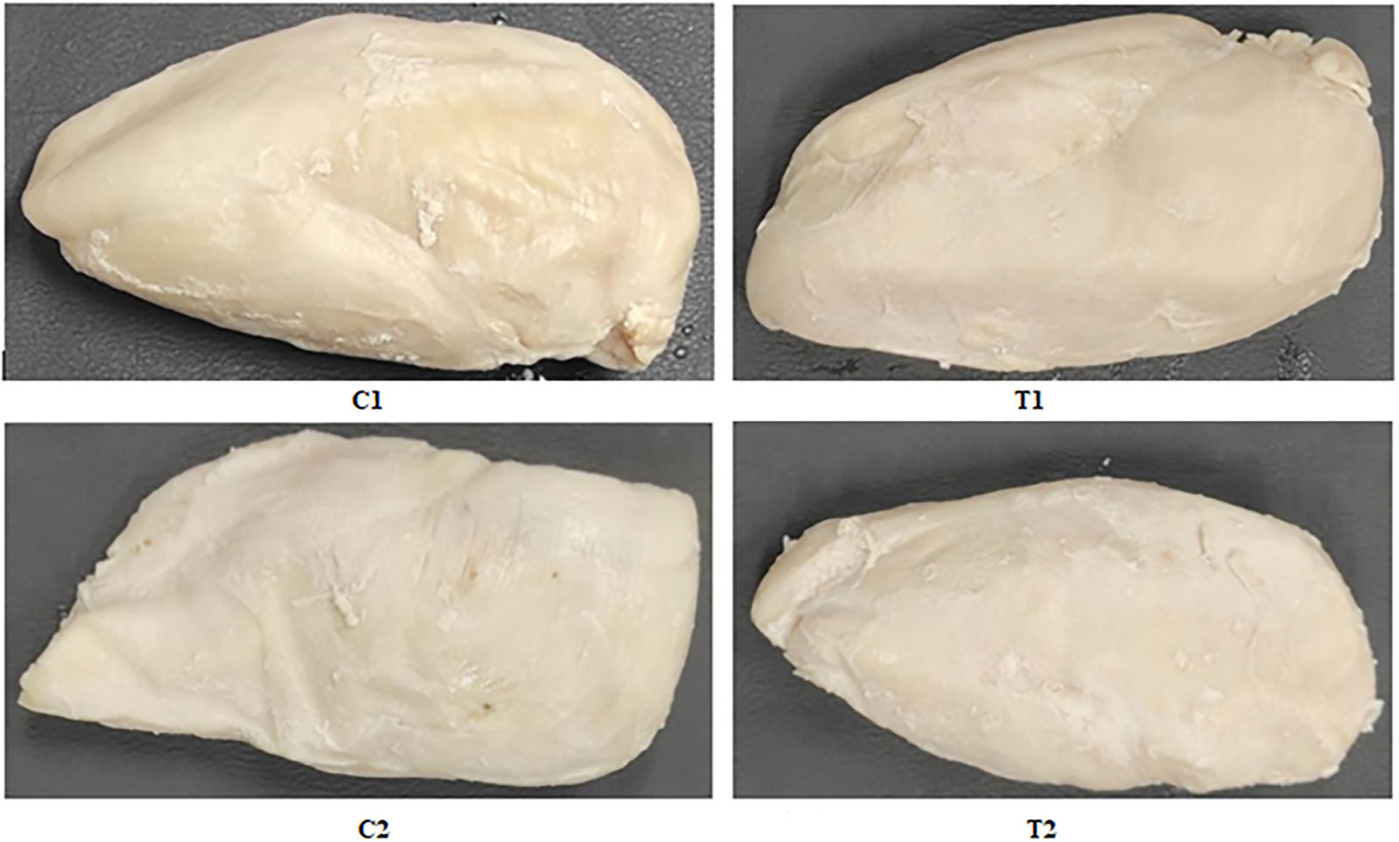
Visual aspect of marinated chicken breast fillet samples subjected to heat treatments using the sous‐vide technique and their respective controls.

Regarding color parameters, lightness (*L* ^*^) was lower in T2 (Figures 4c and 5), likely due to greater moisture loss at higher temperatures. In contrast, the greater water retention in treatments such as T1 increased light reflectance, resulting in a lighter meat appearance. This effect is associated with the optical properties of water in muscle fibers and its interaction with vacuum conditions (Arvanitoyannis et al. 2011; Sánchez del Pulgar et al. 2012).

Chroma (*C* ^*^) was also significantly affected by cooking time and temperature (Figure 4d), with lower values observed in high‐temperature treatments (C2 and T2). T1 maintained more stable coloration, with a redder and lighter appearance over time compared to controls. This difference may be explained by thermal denaturation of myoglobin, the primary meat pigment, which denatures between 55°C and 65°C and turns brown above 70°C (King and Whyte 2006). In addition, deoxymyoglobin, the predominant pigment in vacuum‐packaged meats, is more heat‐resistant than oxymyoglobin and metmyoglobin (Van‐Laack et al. 1996).

Color difference (Δ*E*) values between treatments revealed variation related to cooking method (vacuum or non‐vacuum) and thermal conditions. The values were 5.47 for C1, 5.16 for T1, 4.78 for C2, and 4.15 for T2. In both time–temperature conditions, vacuum treatments (T1 and T2) showed lower Δ*E* values than their respective controls, indicating less color alteration due to sous‐vide cooking. This may be associated with reduced oxygen exposure during processing, limiting oxidative reactions and non‐enzymatic browning, such as Maillard reactions, thus better preserving natural pigments like myoglobin.

In addition, increasing time and temperature (comparing C1 to C2 and T1 to T2) resulted in lower Δ*E* values, suggesting that more intense thermal conditions promoted more complete and uniform protein denaturation, contributing to a more stable and homogeneous appearance. Although all samples had Δ*E* values above 4, indicating perceptible color changes, the lower Δ*E* values in sous‐vide treatments reflect less intense color alteration compared to controls.

Complementing these findings, Figure 3b presents the PCA based on physicochemical variables monitored during storage (syneresis, aw, pH, acidity, *L* ^*^, *a* ^*^, *b* ^*^, Δ*E*, *C* ^*^, and aerobic mesophilic bacteria). The first two principal components (PC1 and PC2) explained 50.27% of the total variance (30.19% and 20.09%, respectively), indicating low variability among treatments over time. Pearson's correlation analysis showed significant relationships only among color parameters, particularly *L* ^*^ and Δ*E* (*r*  =  0.996), and *b* ^*^ and chroma *C* ^*^ (*r*  =  0.998), indicating strong interdependence of these variables in visual characterization of the products.

These results demonstrate that sous‐vide processing, especially under moderate temperature conditions (T1) combined with vacuum packaging, provides greater physicochemical and visual stability in chicken breast meat, enhancing product quality during refrigerated storage. These findings reinforce the potential of sous‐vide as a promising technique for minimally processed meat products, as described in previous studies (Nazzaro et al. 2013; Chang et al. 2023; Nyam et al. 2023; Park et al. 2020; Przybylski et al. 2021).

Microbiological stability during storage was evaluated through specific analyses targeting indicator and pathogenic microorganisms. For aerobic mesophilic bacteria (Figure 3b), control treatments C1 and C2 reached counts of approximately 2 log CFU/g at Day 45 of refrigerated storage. In contrast, sous‐vide treatments T1 and T2 maintained counts below the detection limit (1 log CFU/g), confirming the efficacy of thermal processing in limiting microbial proliferation over time.

*L. monocytogenes* followed a similar trend. While controls C1 and C2 reached ∼10^2^ CFU/g at 45 days, treated samples T1 and T2 remained below the detection threshold (< 1 log CFU/g). These results confirm the microbiological safety of sous‐vide products, meeting legal limits for ready‐to‐eat foods regarding listeriosis prevention. It is worth noting, however, that the presence of *L. monocytogenes* in heat‐treated products may result from post‐process cross‐contamination, as reported by Jordan and McAuliffe (2018) in cooked breaded chicken and refrigerated poultry, which were linked to sporadic listeriosis cases.

Analyses for *S. aureus*, *E. coli*, and *C. perfringens* also showed levels below detection limits in all samples, and *Salmonella* spp. was absent in both control (C1 and C2) and treated groups (T1 and T2) at the end of storage. These results further support the effectiveness of the thermal treatment applied, especially when considering the known thermal resistance of these pathogens.

Thus, the results confirm the microbiological quality and food safety of sous‐vide processed products, fully complying with microbiological standards established by current regulations (Brazil 2022a).

In addition to enhancing technological and sensory attributes, the sous‐vide process offers potential sustainability and efficiency advantages at the industrial level. The higher moisture retention observed in T1 and the reduced syneresis across treatments indicate improved yield, which may translate into better raw material utilization and reduced product loss. The very low peroxide values (< 0.5 mEq O_2_/kg) and microbiological stability over 45 days of refrigerated storage also suggest an extended shelf life, potentially decreasing waste along the distribution chain. Moreover, the precise temperature control characteristic of sous‐vide systems favors more efficient heat transfer compared with conventional cooking, with potential reductions in energy consumption and in the need for added antioxidants or preservatives. Together, these aspects highlight the technological, economic, and environmental relevance of adopting sous‐vide at industrial scale.

## 4 Conclusion

The industrial validation of sous‐vide cooking confirms its readiness for direct application in commercial poultry processing, bridging the gap between experimental research and large‐scale feasibility. Beyond ensuring regulatory compliance, the technique successfully aligns microbiological safety with consumer‐driven demands for juiciness and tenderness, reinforcing its market potential in the ready‐to‐eat segment. The critical role of vacuum packaging highlights the need to integrate packaging innovations with thermal processing strategies to secure product stability and quality. Texture emerged as a decisive marker, indicating that precise control of time–temperature conditions should be central to the industrial design of sous‐vide processes. Moreover, the demonstrated extension of shelf life offers opportunities to reduce costs in logistics and inventory management, contributing to greater competitiveness. In addition to these benefits, sous‐vide processing may contribute to sustainability and operational efficiency. The lower thermal gradients and precise temperature control inherent to the technique can reduce energy consumption, while higher moisture retention improves yield and decreases product waste during storage. These aspects strengthen the practical relevance of sous‐vide as a resource‐efficient alternative to conventional cooking methods.

## Nomenclature

PET: polyethylene terephthalate
LDPE: low‐density polyethylene
LLDPE: linear low‐density polyethylene
PA: polyamide
OTR: oxygen transmission rate
ASTM: American Society for Testing and Materials
*T*_ref_
: Lethality Reference Temperatures
*D*_ref_
: Decimal Reduction Time
*F*_ref_
: Reference Lethality Value
*F*_cal_
: Accumulated Lethality Rate
TPA: texture profile analysis
AFNOR: Association French for Normalization
PCR: polymerase chain reaction
ANOVA: analysis of variance
PCA: principal component analysis
WHC: water‐holding capacity
CFU: colony‐forming unit

## Author Contributions

**Karem Muraro**: conceptualization, formal analysis, data curation, methodology. **Rogério Luis Cansian**: resources, writing – original draft, methodology. **Geciane Toniazzo Backes**: writing – review and editing, supervision. **Eunice Valduga**: investigation, supervision, resources, writing – review and editing, conceptualization.

## Conflicts of Interest

The authors declare no conflicts of interest.
